# Short-Term Spirulina (*Spirulina platensis*) Supplementation and Laying Hen Strain Effects on Eggs’ Lipid Profile and Stability

**DOI:** 10.3390/ani11071944

**Published:** 2021-06-29

**Authors:** Ana I. Rey, Almudena de-Cara, Almudena Rebolé, Ignacio Arija

**Affiliations:** Departamento Producción Animal, Facultad de Veterinaria, Universidad Complutense de Madrid, Avda. Puerta de Hierro s/n., 28040 Madrid, Spain; almudeca@ucm.es (A.d.-C.); arebole@ucm.es (A.R.); arijai@ucm.es (I.A.)

**Keywords:** *Spirulina platensis*, egg colour, retinol, vitamin E, yolk fatty acids, hen strain

## Abstract

**Simple Summary:**

Spirulina is an alga rich in micronutrients of interest for improving the nutritional value and quality of the products. This study evaluates the transfer of some of these micronutrients (vitamins and fatty acids) in egg yolk when short periods of supplementation are used in hens of two different strains, and its consequent effects on some quality parameters. Short-term spirulina supplementation markedly modified vitamin content and color in yolk. Hen strain mainly affected the fatty acid profile, and a different response to retinol content and fatty acid proportions (mainly monounsaturated and desaturase activity) according to spirulina supplementation and breed of hens was observed.

**Abstract:**

The aim of the study was to investigate the effect of short-term dietary spirulina supplementation (1% and 3%) and the strain of laying hens (White Leghorn: WL and Rhode Island Red: RIR) on color, nutritional value, and stability of yolk. Egg weight was not affected by any of the studied effects. Yolks from 3%-spirulina supplemented hens had higher retinol and lower α-tocopherol content (*p* = 0.0001) when compared to control. The supplementation with 1%-spirulina markedly decreased luminosity and increased redness (*p* = 0.0001) and yellowness (*p* = 0.0103). Short-term spirulina supplementation slightly modified the fatty acid composition of yolk. The C16-desaturase index increased with the algae, whereas other egg quality indices (hypocholesterolemic, thrombombogenic, n-6/n-3) were not modified. Hen strain mainly affected to the lipid profile. The RIR hens accumulated greater yolk retinol with supplementation doses of 3% (*p* < 0.05), while the WL hardly suffered changes in the accumulation. Also, yolks from RIR hens had lower C16:0 (*p* = 0.0001), C18:0 (*p* = 0.0001), saturated (SAT) (*p* = 0.0001), and thrombogenic index (*p* = 0.0001), C20:3n-6 (*p* = 0.0001), n-6/n-3 ratio (*p* = 0.003), Δ-6+5-desaturase (*p* = 0.0005), total elongase indices (*p* = 0.0001) when compared to WL. Moreover, RIR had higher monounsaturated (MUFA), Δ-9-desaturase and hypocholesterolemic indices (*p* < 0.05) than WL. A different response to spirulina supplementation was observed for C18:1n-9, MUFA, Δ-9-desaturase and thiesterase indices (*p* < 0.05) according to hen strain. Yolks from RIR had higher MUFA and Δ-9-desaturase indices than WL at 1%-spirulina supplementation, whereas these parameters were less affected in RIR supplemented with 3%. SAT and Δ-9-desaturase were significantly correlated (r = −0.38 and 0.47, respectively) with retinol content according to a linear adjustment (*p* < 0.05). Lipid oxidation of yolk was slightly modified by the dietary treatment or hen strain. It was detected a relationship between TBARS and α-tocopherol, C22:5n-3 or C22:6n-3 (*p* < 0.05). L* and a* were also inversely or positively related with yolk retinol content according to a linear response (*p* < 0.05). The administration of 1% of spirulina in diets of red hens would be an interesting alternative to get healthier eggs from the nutritional point of view, obtaining an adequate color and without modifications in other yolk quality traits.

## 1. Introduction

Spirulina (*Spirulina platensis*) is a blue-green microalgae that has been used as a food source for a long time [1]. One of spirulina′s main points of interest in relation to nutrition is that it contains a very high amount of protein when compared with other foods, providing most of the essential amino acids [2]. Spirulina is also a good source of carotenoids [1], making it an additive of interest in animal production systems such as poultry and fish [3] where obtaining good pigmentation of the product is a key aspect for the consumer. Spirulina has therefore been used at low doses for periods of around 3–24 weeks to improve yolk color [4,5,6,7]. It is also a good source of other micronutrients, mainly vitamins and minerals, containing 3100% more β-carotene than carrots, and is considered a good source of vitamin A [1], vitamin E and other phenolic compounds [8]. Spirulina also has 5100% more iron than spinach [1]. This microalga provides a low amount of lipids but is rich in γ-linolenic acid (C18:3n-6, GLA) [9], which is considered an n-6 with anti-inflammatory properties, playing an important role in treating chronic diseases [10]. In addition, some studies indicate that this microalga has many other benefits for health, such as in vivo antioxidant power, cardiovascular disease protection, hypolipidemic and neuroprotective effects [2,11].

Spirulina’s high nutrient content and beneficial effects on consumer health have made this alga one of the possible ingredients for trying to improve the nutritional quality of animal products. In poultry, some studies have reported its effectiveness in reducing egg cholesterol [5,12] or modifying other egg quality parameters such as shell thickness [7]. However, there is little to no information on spirulina’s effects on the composition of micronutrients such as antioxidant vitamins, the specific fatty acid proportion or how spirulina supplementation may affect products’ lipid stability. The little information available indicates that 5%-spirulina supplementation in poultry diets for a long period may reduce saturated fatty acids and increase monounsaturated fatty acids in egg yolks [13], reducing lipid oxidation. Meanwhile, other authors have reported an increase in unsaturated fatty acids in 0.3% spirulina feeding [14]. In addition, it has been observed that it is possible to modify egg yolks’ vitamin content through dietary modifications, with the transfer of vitamin A being higher than that of other fat-soluble vitamins [15]. Since spirulina is a good source of vitamin A, especially in its provitamin form [1], and since this can compete with vitamin E [16,17], which is deposited in cell membranes, exerting its antioxidant activity in vivo or on the stability of the products [18,19], the possible changes in accumulation due to spirulina supplementation is an aspect of interest that is worth studying.

Spirulina has reached a high price on the market, limiting its use in animal feeding [20]. This makes trying to use short administration times to achieve positive effects a key goal. It has been reported that improvement in yolk color (through carotenoid accumulation) is effective after 1 week of 1.5%-spirulina supplementation in hen diets [7]. However, the effects on the nutritional composition of the egg or other quality parameters with short periods of supplementation are unknown.

There is also a lack of information about the possible effects of this algae on different hen strains. Taking into account that strain may affect egg quality [21,22], the study of this possible varied response to spirulina supplementation on lipid composition and quality of eggs according to breed deserves more attention.

Therefore, the objective of this study was to investigate the effect of short-term dietary spirulina supplementation (1% and 3%) and the strain of laying hens (White Leghorn vs. Rhode Island Red), as well as the interaction between the two, on color and nutritional value (measured as the fatty acid profile and vitamin content), and how these changes may affect yolk stability.

## 2. Materials and Methods 

### 2.1. Animals, Experimental Diets and Sample Collection

The study was conducted at the environmentally controlled farm facility of Faculty of Veterinary Science (Universidad Complutense of Madrid, Spain). All the experimental procedures performed in this study complied with Spanish policy for Animal Protection (RD 53/2013) [23] and farm establishments had approved registration (approval number ES280790000213) and were provided with the facilities for protection and wellness of laying hens (RD 3/2002) [24]. The study only involved egg collection in a nutritional trial without any additional practices or direct handling of animals than those normally adopted during breeding.

Fifty-four hens (half White Leghorn and half Rhode Island Red) of 23 weeks of age with an average weight of 1940 ± 40 g were used. Hens were located in cages (half white and half red in each cage) with a minimum of 550 cm^2^ per hen, provided with perchs, two drinkers and a nest area. Hens received the same diet formulated according to NRC recommendations [25] during 3 weeks prior to the experimental period. For the experiment, 3 cages per treatment were used (a total of 9 cages, 6 animals per cage, 3 per strain and cage) so each treatment was replicated 3 times. During the experimental phase (15 days), ambient temperature was maintained at 20 °C and light was switched off for 8 h/day. Animals were fed with a basal diet formulated according to NRC [25] requirements for hens and was identical for all the experimental groups except for the supplemented spirulina (*Spirulina platensis*) that was administered at 1% or 3% (Table 1). One group without spirulina supplementation acted as control. Spirulina was provided by HealthyTree Company (Oxfordshide, UK) (Table 1). Diets were isoproteic and isoenergetic. Food and water were provided *ad libitum*.

After 15 days of feeding the experimental diets, eggs were collected on days 15 and 16 (*n* = 54; *n* = 18 per experimental treatment; *n* = 27 per strain) and were weighed. Then, yolk was separated, instrumental color was measured, and a sample was taken for lipid oxidation quantification within 48 h of collection. The remaining yolk was kept at −20 °C for the other analysis determinations (fatty acid profile, vitamins quantification) that were carried out before 1 month.

### 2.2. Laboratory Analysis

#### 2.2.1. Extraction and Quantification of Total Fat and Fatty Acid Profile of Feed Samples

Fatty acids of diet were extracted and quantified using the one-step procedure described by Sukhija and Palmquist [26] in lyophilized samples. Pentadecenoic acid (C15:1) (Sigma, Alcobendas, Madrid, Spain) was used as the internal standard. Previously, methylated fatty acids samples were identified according to Rey et al. [19] using a gas chromatograph (Model HP6890; Hewlett Packard Co., Avondale, PA, USA) and a 30 m × 0.32 mm × 0.25 μm cross-linked polyethylene glycol capillary column (Agilent Technologies GmbH, Germany). A temperature program of 170 °C to 245 °C was used. The injector and detector were maintained at 250 °C. The carrier gas (nitrogen) flow rate was 3 mL/min.

#### 2.2.2. Instrumental Color Analysis.

Yolk color was evaluated by means of a chromameter (CM 2002, Minolta, Camera, Osaka, Japan) previously calibrated against a white tile in accordance with the manufacturer’s recommendations [27]. Conditions for measurement (according to CIE system) were D65 illuminant, observer 2 in SCI mode and 1 cm aperture. The average of three random readings was used to measure lightness (L*), redness (a*) and yellowness (b*). In addition, the chroma and hue angle were calculated as chroma = (a*^2^ + b*^2^)^0.5^ and hue = arctg (b*/a*) respectively [25].

#### 2.2.3. Iron-Induced Lipid Oxidation of Yolk Samples.

The liability of yolk homogenates to iron-ascorbate-induced lipid oxidation was determined by a modification of the method of Kornbrust and Mavis [28]. Homogenates (approximately 1 mg protein/mL buffer) were incubated at 37 °C in 40 nM Tris-maleate buffer (pH 7.4) with 1 mM FeSO_4_ in a total volume of 10 mL in duplicated samples. At fixed intervals (0, 30, 60, 90 and 120 min), 0.4 mL were removed for measurement of 2-thiobarbituric acid-reactive substances (TBARS). Absorbance was measured spectrophotometrically at 532 nm (ScanGo, ThermoFisher Scientific, Alcobendas, Spain). TBARS were expressed as nmol malondialdehyde (MDA)/kg yolk. A standard curve was built with 1,1,3,3-Tetraethoxipropane.

#### 2.2.4. Extraction and Quantification of Vitamins E and A in Feed and Yolk Samples

Yolk samples were mixed with a buffer (0.054 M dibasic sodium phosphate) adjusted to pH 7.0 with HCl and absolute ethanol. To extract vitamins (tocopherols and retinol) hexane was added to the mixture and after mixing and centrifugation (600× *g* during 10 min at 4 °C), the upper layer containing vitamins was collected [29]. The upper layer was then evaporated to dryness and dissolved in ethanol prior to analysis by High Performance Liquid Chromatography (HPLC). In feed, α-tocopherol concentration was quantified by sample saponification in the presence of KCl (1.15%) and KOH (50%) [29]. Tocopherols and retinol were analyzed in duplicate by reverse phase HPLC (HP 1100, equipped with a diode array detector and a RP-C18 column) (Agilent Technologies, Waldbronn, Germany). Identification and quantification (µg of α-tocopherol per g and µg of retinol per g of yolk) [16,29] were carried out by means of a standard curve built using the pure compounds (Sigma Aldrich, Alcobendas, Madrid, Spain).

#### 2.2.5. Extraction and Quantification of Total Fat and Fatty Acid Profile of Yolk Samples

Lyophilized yolks were weighed in a safe-lock micro test tube, and after the addition of a solvent mixture (1.5 mL dichloromethane-methanol 8:2) were homogenized in a mixer mill (MM400, Retsch technology, Haan, Germany). Then, the mixture was centrifuged (8 min at 10,000 rpm) and after collection of the upper layer this was evaporated under nitrogen stream [30]. The lipid content was gravimetrically determined. Then, lipids were esterified by heating (80 °C for 1 h) in presence of methanol: toluene: H_2_SO_4_ (88:10:2 by volume) as described elsewhere [19] and the fatty acids methyl esters (FAMEs) were extracted with hexane. FAMEs were separated in a gas chromatograph (HP 6890 Series GC System; Hewlett Packard, Avondale, PA) after direct injection of the sample. Injector temperature was hold at 170 °C. The gas chromatograph was equipped with flame ionization detector and a column HP-Innowax polyethylene glycol (30 m × 0.316 mm × 0.25 µm). After injection, the oven temperature raised to 210 °C at a rate 3.5 °C/min, then to 250 °C at a rate of 7 °C/min and was held constant for 1 min. Detector temperature was held at 250 °C. Identification and quantification of the FAMEs were made by comparing the retention times with those of authentic standards (Sigma–Aldrich, Alcobendas, Spain). Results were expressed by g per 100 g of quantified fatty acids.

Different indices were measured to estimate desaturase or elongase activities or quality. Hypocholesterolemic and Thrombogenic index were calculated as follows [31]:

Hypocholesterolemic index = (C18:1 + C18:2 + C18:3 + C20:3 + C20:4 + C20:5 + C22:4 + C22:5 + C22:6)/(C14:0 + C16:0)(1)

Thrombogenic index = (C14:0 + C16:0 + C18:0)/(0.5 × ∑MUFA + 0.5 × ∑n-6 + 3 × ∑n-3 + ∑n-3/∑n-6)(2)

Δ9 − desaturase index was calculated with the following equation:

Δ9 − desaturase index = (C14:1 + C16:1 + C18:1)/C14:0 + C14:1 + C16:0 + C16:1 + C18:0 + C18:1).(3)

The specific Δ9 − desaturase index was calculated as the ratio of the monounsaturated fatty acid to the sum of the monounsaturated fatty acid plus the saturated fatty acid of the same number of carbons.

Δ6 + Δ5 − desaturase index as an evaluation of the enzymes that participate in the desaturation of C18:2n-6 and C18:3n-3 to their long chain fatty acids and was calculated with the following equation [32]:

Δ5+ Δ6-desaturase = (C20:4n-6 + C20:5n-3 + C22:5n-3 + C22:6n-3)/(C18:2n-6 + C18:3n-3 + C20:4n-6 + C20:5n-3 + C22:5n-3 + C22:6n-3)(4)

The elongase index was calculated as the ratio of C18:0 to C16:0, whereas the thioesterase index was calculated as the ratio of C16:0 to C14:0 [33]

#### 2.2.6. Statistical Analysis

The experimental unit for analysis of all data was the egg. Data were analyzed following a completely randomized design using the general linear model (GLM) procedure contained in SAS (version 9; SAS Inst. Inc., Cary, NC, USA). To study differences in yolk composition, color, TBARS and fatty acid proportions, dietary treatment and strain were considered the fix effects.

Data were presented as the mean of each group and the standard error of the mean (SEM) together with significance levels (*p* value). Duncan′s test was used to separate treatment means. Differences between means were considered statistically significant at *p* < 0.05. Pearson correlation analyses were carried out among total MDA concentration and fat percentage of yolk and different compounds (vitamins and fatty acids) using Statgraphics-18 program. Linear adjustment between variables was carried out by means of Statgraphics-18 program.

## 3. Results

The analyzed fatty acid profile and vitamin E content of the diets is presented in Table 2. The main changes in fatty acid profile by the spirulina supplementation were found in the proportion of C16:0, C18:3n-6 (γ-linolenic acid), total saturated fatty acids and α-tocopherol content that increased with the spirulina supply.

Egg weight was not affected by the spirulina supplementation or the hen strain (Table 3). However, the yolk composition was modified by the short-period of either the spirulina supplementation or the hen strain. Hence, yolks from hens supplemented with 3% spirulina had higher (*p* = 0.0023) retinol and lower (*p* = 0.002) α-tocopherol content, when compared to control group; whereas 1%-spirulina supplemented group had intermediate values. No changes were detected in yolk fat or γ-tocopherol content by the short-term dietary spirulina supplementation. The hen strain also affected on the yolk composition. RIR hens had higher fat (*p* = 0.001) that resulted in changes in yolk humidity. Moreover, RIR hens had higher α-tocopherol and retinol contents in yolk than WL (*p* = 0.0001 and 0.0001, respectively). An interaction was found according to spirulina supplementation and hen strain on yolk retinol content.

Color changes in egg yolks according to short-term of spirulina supplementation is shown in Table 4. 1%-Spirulina supplementation markedly decreased (*p* = 0.0001) luminosity (L value) and increased (*p* = 0.0001) redness (a* value) and yellowness (b* value). The intensity of color (chroma) and a*/b* ratio also increased with spirulina supplementation (*p* = 0.0001), whereas tone (hue value) decreased (*p* = 0.0001). Eggs from group receiving 1%-spirulina supplementation showed intermediate values. Hue angle value also increased (*p* = 0.0001) in those supplemented groups when compared with control. Less changes in color were observed according to the hen strain. L* was lower (*p* = 0.0003) and the ratio a*/b* higher (*p* = 0.03) in RIR than in WL.

The fatty acid composition of the yolk according to spirulina supplementation or hen strain is presented in Table 5 and Table 6. Short-term spirulina supplementation slightly modified the fatty acid composition of yolk. Hence, yolks from 1% or 3%-supplemented hens had lower C16:1n-9 (*p* = 0.0001) and C22:6n-3 (*p* = 0.0047) proportions when compared to the control group; meanwhile yolks from 3%-supplemented hens had higher C17:0, C17:1 (*p* = 0.0001) when compared to the other groups. The main fatty acids (saturated, monounsaturated and polyunsaturated) of yolk were not modified by the spirulina supplementation in the diet. C16-desaturase index increased with the algae supplementation, whereas other egg quality index (hypocholesterolemic, thrombombogenic, n6/n3) were not modified.

The main changes in fatty acid composition were detected by the hen strain effect. Yolks from WL had lower proportions of C16:1n-9 (*p* = 0.0001), C17:0 (*p* = 0.0001), C17:1 (*p* = 0.0001), C18:1n-7 (0.0005), C18:2n-6 (*p* = 0.0001), C18:3n-3 (*p* = 0.0001), C18:4n-3 (*p* = 0.0001), C22:4n-6 (0.0001), C22:5n-3 (*p* = 0.0055), and C22:6n-3 (*p* = 0.0056) that resulted in lower polyunsaturated fatty acids (PUFA) (*p* = 0.0001), n-6 (*p* = 0.0001) and n-3 (*p* = 0.0001) when compared to RIR. However, RIR had lower C16:0 (*p* = 0.0001), C18:0 (*p* = 0.0001), total saturated (SAT) (*p* = 0.0001), thrombogenic index (*p* = 0.0001), C20:3n-6 (*p* = 0.0001), n-6/n-3 ratio (*p* = 0.003), Δ-6+5-desaturase index (*p* = 0.0005), total elongase and C18/C16-elongase indices (*p* = 0.0001) when compared to WL. Moreover, RIR had higher monounsaturated fatty acids (MUFA) (*p* = 0.05) and hypocholesterolemic index (*p* = 0.0026) than WL. The desaturase, elongase and thiesterase indicators were also modified by hen strain. RIR had higher total Δ-9-desaturase index (*p* = 0.0001), C14-desaturase (*p* = 0.0107), C16-desaturase (*p* = 0.0001), C18-desaturase (*p* = 0.0001) and C20-desaturase (*p* = 0.0036) than WL.

**Figure 1:**
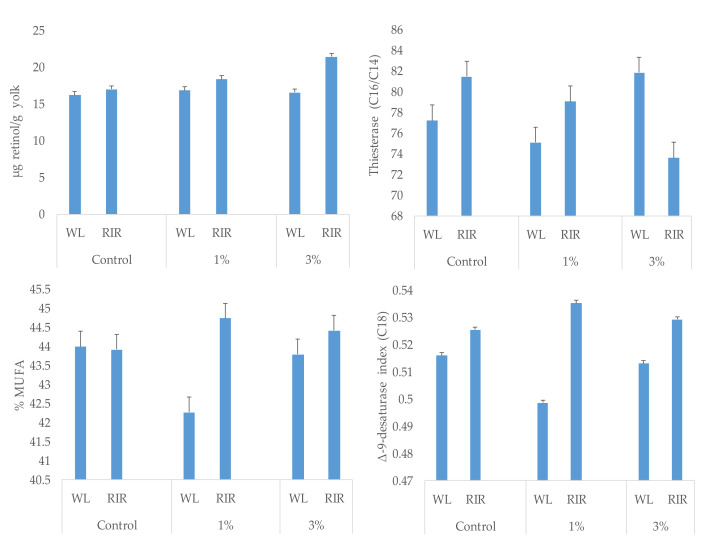
Interaction effect (*p* < 0.05) in yolk retinol content, monounsaturated fatty acids (MUFA) proportion, Δ9-desaturase and thiesterase indices according to spirulina supplementation and hen strain (White Leghorn: WL or Rhode Island Red: RIR).

A different response to spirulina supplementation was observed for C14:0 (*p* = 0.0047), C18:1n-9 (*p* = 0.0248), C22:5n-3 (*p* = 0.0052), MUFA (*p* = 0.0309), Δ-9-desaturase index (*p* = 0.0271) and thiesterase index (*p* = 0.0097) according to hen strain (interaction effect) (Figure 1).

Lipid stability of yolk according to dietary treatment or hen strain is presented in Table 7. Little changes were observed on lipid oxidation by short-term spirulina supplementation. Malondialdehyde (MDA) concentration was higher at the initial time of incubation (*p* = 0.0209) in those spirulina-supplemented groups, whereas statistical differences were not observed by times 60, 90, and 180 of incubation. Strain of hens neither affected the lipid stability of yolks; however, at 90 min of iron-induced incubation, RIR hens tended to have higher (*p* = 0.0947) MDA content in yolk when compared to WL.

The relation between some quality traits and fatty acids or vitamin composition of yolks is presented in Table 8. Total TBARS and α-tocopherol contents were inversely related (r = −0.42; R^2^ = 17%; *p* = 0.0125) and fixed to a linear response. A similar linear but positive relationship was observed between total TBARS and C22:5n-3 (r = 0.39; R^2^ = 15%; *p* = 0.025) or C22:6n-3 (r = 0.38; R^2^ = 15%; *p* = 0.026). Moreover, L* and a* values were inversely or positively related respectively, with yolk retinol content (r = −0.46, R^2^ = 21%; *p* = 0.0012 and r = 0.49, R^2^ = 23%, *p* = 0.0015). Some fatty acids or their indices were also correlated with retinol content. So, SAT, C18:0 and elongase were inversely correlated with retinol (r = −0.38; −0.52 and −0.51, respectively); whereas delta-9-desaturase index was positively correlated (r = 0.47). Finally, the fat proportion was also inversely related with yolk humidity (r = −0.72, R^2^ = 51%, *p* = 0.0001).

## 4. Discussion

The inclusion of spirulina into diets mainly modified the proportions of C16:0, C18:3n-6 (γ-linolenic acid) that increased 17% and 3500% respectively in the 3%-supplemented diet when compared to the basal control diet. Other fatty acids such as C22:5n-3 and C22:6n-3 suffered a 13% increase in the diet with the highest spirulina supplementation. Spirulina is a good source of these fatty acids, as stated by other authors [9]. Algae inclusion in diets also increased the content of α-tocopherol in the feed by up to 48%, which reflects other authors’ findings [2,8,34]. This interesting nutrient composition of diets containing spirulina would be expected to be transferred into the egg, however, since spirulina is an expensive product, we checked short supplementation times in which egg color could be modified.

Egg weight was not significantly affected by the dietary inclusion of 1% or 3% spirulina for 15 days of supplementation. Other investigations using doses of 1–2% for a longer period (12 weeks) did not find changes in this parameter [12]. Omri et al. [7] reported that doses of 1.5% and at least 6 weeks were necessary for changes in egg weight to be observed. Of the differences observed in the literature, the short period used in the present study seemed to be insufficient to affect this parameter.

In terms of yolk composition, since spirulina is a poor source of fat [9], its supplementation into diets did not modify the total fat proportion of yolk. However, liposoluble vitamins underwent some changes. It is interesting to note that 3% spirulina supplementation increased yolk retinol that resulted in lower α-tocopherol content when compared with the control group. However, 1% supplementation for 15 days was not enough to induce significant changes in vitamin content. Spirulina provides a very high amount of β-carotene [1], which is converted into retinol (sometimes in the place of absorption) [35], being considered a good source of vitamin A [1]. Spirulina also provides vitamin E [8]. However, there is no information in the literature concerning modifications to the accumulation of vitamins, such as tocopherols in yolk, through spirulina supplementation. In chicken meat, 15% spirulina supplementation resulted in a lower accumulation of vitamin E [36]. The competence between these two liposoluble vitamins has been widely reported in the literature [16,37]. Grobas et al. [17] found that hens supplemented with the highest level of vitamin A in their diets had lower α-tocopherol concentration in their yolks. A similar effect was found by Jiang et al. [38], when diets were enriched with β-carotene, and Surai et al. [39], when hens were supplemented with vitamin A for 28 weeks. Naber et al. [15] found a higher transfer of dietary vitamin A (around 70%) into the yolk than other liposoluble vitamins such as vitamin E (20–30%). The results of the present study indicate that short-term supplementation at doses of 1% spirulina are more adequate in order to avoid reducing the levels of vitamin E in the yolk, since this vitamin has important antioxidant functions in the body [18,19,40]. Another interesting result of the present experiment is that the accumulation of retinol and α-tocopherol in the yolk depended not only on the dose of spirulina supplementation in the diet but also on the breed of hens. This meant that RIR accumulated more vitamins in the yolk than WL in relation to the higher fat content. Moreover, an interaction effect was observed, and RIR hens accumulated greater yolk retinol with supplementation doses of 3%, while the WL hens hardly underwent any changes in accumulation. The transformation of carotenes into vitamin A relies on the presence of the converting enzyme, although differences in the accumulation range have also been described depending on the rusticity of the birds [35]. 

The daily recommendations for these vitamins are between 800–1000 µg and 10,000 µg for vitamins A and E respectively in people between 10 and 60 years old [41]. This means that the ingestion of an egg from a hen supplemented with 1% spirulina would provide 50% or 42% of the recommended daily values for vitamins E and A, respectively. However, the final accumulation in yolk depends on the liver accumulation of hens [37] or the supplementation of vitamin premix in the feed, which often includes higher doses than the basic requirements. Attention should therefore be paid in supplementation doses when using a rich source of vitamins such as spirulina microalgae, in order to avoid a decrease in vitamin E.

Short-term spirulina supplementation also markedly modified yolk color, mainly redness that changed from 2.3 in control (corn-based diet) to 6.2 in the 1%-supplemented group. Yellowness was also modified but to a lesser extent (from 32.7 in control to 37.8 in 1%-supplemented). Anderson et al. [4] reported that 1% spirulina supplementation for 3 weeks was enough to improve yolk color according to consumers’ demand. These authors also found changes in color after 4–5 days of supplementation. Other authors [12] also found that the yolk color score improved from 1% doses, although these authors fed hens for a longer period of 12 weeks. Other research using a shorter period (6 weeks) [42] found that differences were not marked between 2 and 2.5% supplementation, with yolks having a pleasing color with 2% supplementation. The authors of that study used a visual scale. In a more accurate study in which color was measured by instrumental means [7], differences in color were detected after 1 week and 1.5% supplementation. RIR hens also had yolks with a higher luminosity, tone and redness/yellowness ratio. Other authors also reported the significant influence of genotype on color [43]. Moreover, redness was directly related with yolk retinol content in the present study. Other authors in the literature [38] reported a linear relationship between yolk retinol and β-carotene given in diets. This is because most of the β-carotene is converted to retinol and transferred to the yolk (only 1% or less of carotene accumulates in the yolks of hen breeds used in intensive breeding) [35].

Although color and vitamin content were modified by the 15d supplementation period, few changes were detected in the fatty acid proportions. The γ-linolenic acid to which important functions have been attributed [10] was not modified in yolks by the supplementation of diets with 1 or 3% spirulina. Only the proportions of C16:1n-9 and C18:1n-7 decreased in supplemented groups, along with C22:6n-3 in 3% supplemented groups, while C17:0 and C17:1 increased in 3% supplemented groups when compared to the control. There is not much information on the possible effects of spirulina supplementation on the fatty acid composition of yolk. Some authors using much higher doses of 5% and longer times detected increases in monounsaturated fatty acids and decreases in polyunsaturated fatty acids [13], while other researchers using similar doses reported increases in linoleic and linolenic acids [44]. In other studies, supplementation times of over 30 days produced increases in the levels of polyunsaturated fatty acids. More information is needed in relation to fatty acid modifications. The results of the present study indicate that longer supplementation times or higher doses would possibly be necessary to increase the content of some interesting fatty acids such as C18:3n-6.

In relation to strain effect, the lipid profile was markedly affected. The red strain resulted in a more nutritionally interesting profile. RIR had lower levels of saturated fatty acids (mainly C16:0 and C18:0) and higher levels of polyunsaturated fatty acids (mainly C18:2n-6 and C18:3n-3), while other interesting fatty acids such as C18:4n-3, C20:5n-3 and C22:6n-3 were also higher in the red hens when compared with the white hens. This resulted in higher desaturase, hypocholesterolemic and thrombogenic indices in RIR hens. Other authors have found that the hen strain influences yolks’ fatty acids [21,45]. When comparing Dekalb Delta Leghorn to Isa Brown [21], the white strain had a higher level of saturated fatty acids and a lower level of monounsaturated fatty acids. Other authors also reported higher saturated, mainly stearic acid, accumulation in Hyline White Leghorn when compared to other lines like Babcock or Dekalb Delta [46], which could be explained by different metabolic patterns. Thus, in the present study, RIR had higher delta-9-desaturase resulting in the formation of monounsaturated fatty acids from saturated fatty acids, while a lower elongase potential was found in RIR than WL. Ayerza and Coates [47] also found an enzyme-limiting capacity according to the strain of hens. The use of a high-n-3 fatty acid diet resulted in different arachidonic acid accumulation in yolks depending on the diet and strain due to their different capacities to utilize desaturase and elongase enzymes. RIR hens also had higher fat content in their yolks and a consequently higher content of liposoluble vitamins. These results would again indicate the different metabolic utilization of fats and fatty acids for energy supply according to breed, as reported by other authors [21,46], which would influence the final accumulation of these nutrients in egg yolks.

Fatty acid proportions were modified in a different manner according to spirulina dose supplementation and hen strain (significant interaction effect), as was yolk retinol content. So, yolks from RIR hens had higher MUFA proportions and Δ-9-desaturase index than WL at 1%-spirulina supplementation, while these parameters were less affected in RIR supplemented with 3%-spirulina. To our knowledge, there is a lack of information on the possible different effects of spirulina supplementation according to strain. Mariey et al. [5], using four levels of spirulina for 24 weeks with two breeds of local hens (Sinai and Gimmizah), did not report any effect on parameters such as egg weight or color, although lipid profile (except cholesterol content) was not studied. There is previous information on pigs that indicates that dietary vitamin A supplementation and changes in fat retinol accumulation modifies the saturated and monounsaturated fatty acids and the enzyme desaturase capacity of tissues [48]. Other authors also found higher stearoyl-CoA desaturase activity in sheep supplemented with vitamin A [49] and a different response to dietary vitamin A according to genotype of pigs and rats or the possibilities of fat accumulation [50,51]. In addition, other antioxidant vitamins such as vitamin E have been linked to higher protection on desaturase and elongase enzymes [40,52]. Changes in vitamin content observed in the present study according to spirulina dose and strain may therefore explain the different response to fatty acid modification in tissues. This is confirmed with the linear and significant relationship found between retinol and saturated fatty acids (mainly C18:0) and Δ-9-desaturase and elongase indices.

The lipid stability of yolk was very slightly modified by the short supplementation of 1 or 3% spirulina. A significant increase in lipid oxidation by spirulina supplementation was only observed in the initial time and no significant changes were detected afterwards, although numbers in supplemented groups were greater. This result is in accordance with the decrease in the concentration of α-tocopherol observed in the yolks from hens supplemented with spirulina. This relationship followed a linear adjustment in the present study. Vitamin E (mainly in the form of α-tocopherol) is considered one of the main antioxidants in vivo and its location in cell membranes allows it to exert interesting antioxidant functions in food products [19,40]. In the present study, a positive relationship between lipid oxidation (measured as TBARS) and long-chain fatty acids such as C22:5n-3 and C22:4n-6 was also observed. These unsaturated fatty acids are highly susceptible to undergoing oxidation reactions [40,53]. Even though significant changes in those fatty acids were not detected in the present study, the numbers of these fatty acids were greater in the spirulina-supplemented groups. Other authors have reported increases in DPA (22:5n-3) and other long-chain fatty acids in eggs through long-term spirulina microalgae extract supplementation [54]. There is not much information on the effects of spirulina on eggs’ lipid stability. Some investigations found an increased total antioxidant potential in serum from hens supplemented for 12 weeks with 2% spirulina in their diets [12]. In addition, Mirzaie et al. [55] reported its effectiveness in elevating antioxidant status by increasing antioxidant enzymes or reducing MDA in heat-exposed broilers. However, the measurements in these studies were taken from the live animals’ serum/plasma and not from the final product. Studies on the possible effects of spirulina supplementation in animal tissues are scarce. Miranda et al. [8] found lower TBARS data after 2 or 7 weeks of treatment in the brain homogenates of supplemented rats when compared to the control group. However, TBARS production was similar to control in liver homogenates. Liver as yolk is one of the main reservoirs of vitamin A in its different forms [16], so the lack of differences in these tissues could be attributed to a lower content of other antioxidant vitamins, as observed in the present study. Conversely, the unique investigation on lipid stability carried out in eggs [13], reported lower lipid peroxidation levels in yolks from quails fed at least 5% spirulina for 42 days. However, these authors also found lower proportions of n-3 fatty acids, which are very susceptible to oxidation [40]. So further information is necessary to determine the effects of this nutrient on yolks’ lipid profile and stability. The results of the present study show that a low dose (1%) of spirulina for a short time could be an interesting alternative in not modifying nutritional value and improving color without modifications in other quality traits.

## 5. Conclusions

In conclusion, the short-term dietary supplementation of spirulina at 1 or 3% modified the α-tocopherol and retinol content of yolk. 1% supplementation would be sufficient to induce positive changes in yolks’ redness and yellowness, without deleterious depletion in α-tocopherol, C22:6n-3 or lipid stability. Hen strain markedly affected yolks’ lipid content, liposoluble vitamins and fatty acid profile, with red line hens having a more favorable profile than white ones from a nutritional and health perspective. In addition, hens responded in a different manner to spirulina according to strain. Longer supplementation times or higher doses of dietary spirulina would probably be necessary to modify the fatty acid profile of the product.

## Figures and Tables

**Table 1:** Composition and calculated major nutrients of the experimental diets ^1^.

Ingredients (%).	Control	1%-SP	3%-SP
Corn grain	63.80	64.60	65.80
Soybean meal (44%CP)	23.60	22.00	18.80
Sunflower oil	1.50	1.30	1.30
Calcium carbonate	8.90	8.90	8.80
Monocalcium phosphate	1.30	1.30	1.30
DL-Methionine	0.10	0.10	0.10
L-Lysine 50	0.00	0.00	0.10
Premix ^2^	0.50	0.50	0.50
Spirulina ^3^	0.00	1.00	3.00
Salt	0.30	0.30	0.30
Vitamin E 50% (dl-α-tocopheryl acetate)	0.04	0.04	0.04
Nutrients (% Total Feed)			
Metabolizable energy (kcal/kg)	2763.50	2763.60	2763.90
Crude protein	16.50	16.50	16.50
Crude fat	3.90	3.80	3.50
Crude fiber	2.80	2.80	2.70
Lys	1.05	1.03	1.03
Met+Cys	0.69	0.69	0.69
Trp	0.19	0.19	0.19
Calcium	3.70	3.70	3.70
Total phosphorous	0.61	0.61	0.60
Available phosphorous	0.37	0.37	0.37
C18:2	2.18	2.12	2.01
Starch	41.9	42.5	43.56

^1^ Diets: 1%-SP: 1%-spirulina supplementation; 3%-SP: 3%-spirulina supplementation.^2^ Premix (content per kg): vitamin A, 8250 IU; vitamin D3, 1000 IU; vitamin E, 11 IU; vitamin K, 1.1 mg; vitamin B12, 11.5 mg; riboflavin, 5.5 mg; Ca pantothenate, 11 mg; niacin, 53.3 mg; choline chloride, 1020 mg; folic acid, 0.75 mg; biotin, 0,25 mg; Mn, 55 mg; Zn, 50 mg; Fe, 80 mg; Cu, 5 mg; Se, 0.1 mg; I, 0.18 mg.^3^ Spirulina nutrient composition (/100 g) (provided by HealthyTree Company, UK): energy, 336 kcal, fiber, 5.1 g, protein, 66 g, sodium, 0.9 g, carbohydrates, 13.1 g, Fe, 6.6 mg, sugar, 0.1 g, vitamin B_12_, 170 µg, fats 1g, saturated fat 0.5 g, Ca 33 mg, Mg 300 mg.

**Table 2:** Fatty acid composition and tocopherol content of the experimental diets ^1^.

Treatments	Control		1%-SP		3%-SP	
	Mean	SD ^7^	Mean	SD	Mean	SD
**Fatty acids (g/100 g total fatty acids)**						
C14:0	0.089	0.007	0.092	0.002	0.124	0.011
C15:1	0.182	0.023	0.137	0.046	0.148	0.002
C16:0	12.184	0.800	12.501	0.105	14.265	0.156
C17:0	0.414	0.044	0.465	0.023	0.463	0.032
C17:1	0.068	0.003	0.075	0.011	0.080	0.001
C18:0	3.149	0.149	2.973	0.041	3.047	0.003
C18:1n-9	25.128	1.133	24.856	0.912	23.815	0.052
C18:1n-7	0.824	0.009	0.866	0.015	0.844	0.010
C18:2n-6	54.935	0.204	54.666	0.668	52.855	0.129
C18:3n-6	0.035	0.001	0.364	0.010	1.264	0.003
C18:3n-3	2.218	0.269	2.183	0.034	2.291	0.036
C20:0	0.400	0.005	0.415	0.010	0.418	0.001
C20:1	0.286	0.019	0.320	0.011	0.302	0.010
C22:4n-6	0.036	0.016	0.031	0.004	0.029	0.000
C22:5n-3	0.027	0.001	0.029	0.002	0.028	0.002
C22:6n-3	0.024	0.004	0.028	0.003	0.028	0.000
∑SAT ^2^	16.235	1.005	16.445	0.180	18.317	0.204
∑MUFA ^3^	26.489	1.186	26.254	0.996	25.189	0.076
∑PUFA ^4^	57.276	0.497	57.301	0.722	56.494	0.170
∑n-6 ^5^	55.006	0.222	55.060	0.682	54.147	0.132
∑n-3 ^6^	2.270	0.275	2.240	0.040	2.347	0.038
**Tocopherols (µg/g)**						
α-Tocopherol	257.118	43.400	279.481	59.637	381.518	59.826
γ-Tocopherol	18.559	6.174	18.014	6.047	14.985	1.781

^1^ Diets: 1%-SP: 1%-spirulina supplementation; 3%-SP: 3%-spirulina supplementation; ^2^ ∑SAT = Sum of total saturated fatty acids; ^3^ ∑MUFA = Sum of total monounsaturated fatty acids; ^4^ ∑PUFA = Sum of total polyunsaturated fatty acids; ^5^ ∑n-6 = Sum of total n-6 fatty acids; ^6^ ∑n-3 = Sum of total n-3 fatty acids; ^7^ SD = Standard deviation (*n* = 2).

**Table 3:** Effect of dietary spirulina and strain of laying hens (White Leghorn: WL or Rhode Island Red: RIR) on egg weight and yolk composition.

Yolk Parameters	Treatment Effect			Strain Effect						
	CONTROL	1%	3%	WL	RIR	SEM ^1^	SEM ^2^	^3^*p* Treatment	*p* Strain	*p* Treatment × Strain
Egg weight, g	61.4	63.1	62.5	61.6	63.1	0.81	0.83	0.3210	0.1107	0.0964
**Yolk composition**										
Humidity, %	51.0	50.4	50.9^A^	51.4 ^A^	50.2 ^B^	0.33	0.33	0.4060	0.0012	0.3475
Fat %	30.6	30.6	29.9	29.9 ^B^	30.9 ^A^	0.32	0.32	0.1706	0.0046	0.5091
α-Tocopherol, µg/g	227.7 ^a^	207.7 ^ba^	168.1 ^b^	165.5 ^B^	236.8 ^A^	11.44	12.27	0.0018	0.0001	0.7099
γ-Tocopherol, µg/g	7.5	6.5	6.5	6.7	6.9	0.38	0.38	0.1417	0.6175	0.5566
Retinol, µg/g	16.6 ^b^	17.6 ^ba^	19.0 ^a^	16.5 ^B^	18.9 ^A^	0.46	0.52	0.0023	0.0001	0.0068

^1^ SEM: standard error of the mean of dietary treatment effect *n* = 18; ^2^ SEM: standard error of the mean of strain effect *n* = 27; ^3^ *p*: Differences were statistically significant when *p* < 0.05. ^a,b,ba,A,B^ Values with different superscript were statistically significant.

**Table 4:** Effect of dietary spirulina and strain of laying hens (White Leghorn: WL or Rhode Island Red: RIR) on color of yolk.

Color Measurements	Dietary Treatment			Strain Effect						
	CONTROL	1%	3%	WL	RIR	SEM ^1^	SEM ^2^	^3^*p* Treatment	*p* Strain	*p* Treatment × strain
CIE L*^4^	61.9 ^a^	59.4 ^b^	56.1 ^c^	60.2 ^A^	58.0 ^B^	0.49	0.67	0.0001	0.0003	0.7554
CIE a*^5^	2.3 ^c^	6.2 ^b^	12.5 ^a^	6.6	7.4	0.38	0.98	0.0001	0.1983	0.4754
CIE b*^6^	32.7 ^b^	37.3 ^a^	36.8 ^a^	35.9	35.3	1.07	0.98	0.0103	0.7228	0.3616
Chroma	32.8 ^b^	37.8 ^a^	38.9 ^a^	36.7	36.3	1.07	1.05	0.0007	0.8008	0.3526
hue	90.2 ^a^	80.7 ^b^	71.1 ^c^	81.7 ^A^	79.6 ^B^	0.86	1.99	0.0001	0.0299	0.1625
hue angle	0.3 ^b^	1.4 ^a^	1.2 ^a^	0.7	1.2	0.23	0.23	0.0033	0.0648	0.0286
a/b	0.1 ^c^	0.2 ^b^	0.3 ^a^	0.2 ^B^	0.2 ^A^	0.02	0.04	0.0001	0.0300	0.1836

^1^ SEM: standard error of the mean of dietary treatment effect *n* = 18; ^2^ SEM: standard error of the mean of strain effect *n* = 27; ^3^ Differences were statistically significant when *p* < 0.05. ^a,b,c,A,B^ Values with different superscript were statistically significant; ^4^ L* (luminosity); ^5^ a* (redness); ^6^ b* (yellowness).

**Table 5:** Effect of dietary spirulina and strain of laying hens (White Leghorn: WL or Rhode Island Red: RIR) on the fatty acid profile (g/100 g total fatty acids) of yolk.

Fatty acids	Dietary Treatment			Strain Effect						
	CONTROL	1%	3%	WL	RIR	SEM ^1^	SEM ^2^	^3^*p* Treatment	*p* Strain	*p* Treatment × Strain
C14:0	0.34	0.35	0.34	0.35	0.33	0.008	0.006	0.7279	0.2578	0.0047
C14:1	0.06	0.06	0.06	0.06	0.06	0.003	0.002	0.8628	0.1246	0.3097
C15:1	0.01	0.01	0.01	0.01 ^B^	0.01 ^A^	0.000	0.000	0.9263	0.0010	0.4418
C16:0	26.33	26.53	26.31	26.91 ^A^	25.87 ^B^	0.210	0.173	0.7570	0.0001	0.0864
C16:1n-9	0.54 ^a^	0.47 ^b^	0.45 ^b^	0.44 ^B^	0.53 ^A^	0.014	0.013	0.0001	0.0001	0.1416
C16:1n-7	2.46	2.51	2.57	2.44 ^B^	2.59 ^A^	0.069	0.055	0.7751	0.0253	0.5226
C17:0	0.17 ^b^	0.18 ^b^	0.20 ^a^	0.17 ^B^	0.19 ^A^	0.003	0.004	0.0001	0.0001	0.2363
C17:1	0.12 ^b^	0.12 ^b^	0.14 ^a^	0.11 ^B^	0.14 ^A^	0.002	0.002	0.0001	0.0001	0.3266
C18:0	9.95	10.00	10.05	10.69 ^A^	9.30 ^B^	0.164	0.133	0.9654	0.0001	0.2591
C18:1n-9	39.18	38.91	39.45	38.89	39.46	0.375	0.317	0.5502	0.3029	0.0248
C18:1n-7	1.27 ^a^	1.12 ^b^	1.13 ^b^	1.09 ^B^	1.26 ^A^	0.040	0.035	0.0089	0.0005	0.1419
C18:2n-6	15.03	15.18	14.78	14.32 ^B^	15.66 ^A^	0.190	0.155	0.3280	0.0001	0.3907
C18:3n-6	0.14	0.15	0.16	0.15	0.15	0.005	0.004	0.2450	0.5470	0.2437
C18:3n-3	0.24	0.24	0.25	0.22 ^B^	0.26 ^A^	0.005	0.004	0.7587	0.0001	0.4910
C18:4n-3	0.07	0.07	0.07	0.07 ^B^	0.08 ^A^	0.002	0.002	0.4446	0.0001	0.1891
C20:0	0.04	0.03	0.04	0.04 ^A^	0.03 ^B^	0.001	0.001	0.0901	0.0065	0.3448
C20:1n-9	0.24	0.23	0.24	0.24	0.24	0.005	0.004	0.2545	0.5548	0.4459
C20:3n-6	0.18	0.17	0.18	0.19 ^A^	0.17 ^B^	0.005	0.004	0.1654	0.0001	0.0602
C20:4n-6	2.57	2.60	2.57	2.60	2.56	0.046	0.038	0.8378	0.3998	0.1817
C20:5n-3	0.04	0.04	0.04	0.05 ^B^	0.04 ^A^	0.001	0.001	0.0937	0.0224	0.1254
C22:1n-9	0.07	0.07	0.07	0.07	0.07	0.003	0.002	0.1448	0.1231	0.1275
C22:4n-6	0.2	0.21	0.22	0.19 ^B^	0.23 ^A^	0.006	0.005	0.2220	0.0001	0.4521
C22:5n-3	0.08	0.09	0.09	0.08 ^B^	0.10 ^A^	0.005	0.005	0.6841	0.0055	0.0052
C22:6n-3	0.67 ^a^	0.65 ^ba^	0.61 ^b^	0.61 ^B^	0.67 ^A^	0.011	0.010	0.0047	0.0001	0.0989

^1^ SEM: standard error of the mean of dietary treatment effect *n* = 18; ^2^ SEM: standard error of the mean of strain effect *n* = 27; ^3^ Differences were statistically significant when *p* < 0.05. ^a,b,ba,A,B^ Values with different superscript were statistically significant.

**Table 6:** Effect of dietary spirulina and strain (White Leghorn: WL or Rhode Island Red: RIR) on the main fatty acids (g/100 g total fatty acids) and different indices of yolk.

Fatty acids	Dietary Treatment			Strain Effect						
	CONTROL	1%	3%	WL	RIR	SEM ^1^	SEM ^2^	*p* Treatment ^3^	*p* Strain	*p* Treatment × Strain
∑SAT ^3^	36.82	37.09	36.93	38.17 ^A^	35.73 ^B^	0.290	0.240	0.9159	0.0001	0.0691
∑MUFA ^4^	43.95	43.50	44.10	43.35 ^B^	44.35 ^A^	0.382	0.322	0.5468	0.0427	0.0309
∑PUFA ^5^	19.19	19.36	18.93	18.44 ^B^	19.88 ^A^	0.212	0.174	0.3453	0.0001	0.2573
∑n-6 ^6^	18.12	18.31	17.91	17.46 ^B^	18.77 ^A^	0.204	0.168	0.3803	0.0001	0.2232
∑n-3 ^7^	1.11 ^a^	1.09 ^ba^	1.06 ^b^	1.02 ^B^	1.15 ^A^	0.015	0.012	0.0539	0.0001	0.7162
∑n-6/∑n-3 ^8^	16.39	16.83	17.01	17.11 ^A^	16.37 ^B^	0.205	0.173	0.1358	0.0034	0.0680
**Indices**										
Hypochol ^9^	2.19	2.17	2.19	2.10 ^B^	2.26 ^A^	0.028	0.023	0.8930	0.0001	0.0629
Thrombogenic ^10^	0.72	0.72	0.72	0.75 ^A^	0.69 ^B^	0.007	0.006	0.7254	0.0001	0.2289
Δ9−desat ^11^	0.52	0.52	0.52	0.51 ^B^	0.53 ^A^	0.004	0.003	0.7648	0.0001	0.0271
Δ9−desat 14 ^12^	0.15	0.14	0.15	0.14 ^B^	0.15 ^A^	0.004	0.003	0.4891	0.0107	0.9931
Δ9−desat 16 ^13^	0.02 ^a^	0.02 ^b^	0.02 ^b^	0.02 ^B^	0.02 ^A^	0.001	0.001	0.0003	0.0001	0.1409
Δ9−desat 18 ^14^	0.80	0.80	0.80	0.78 ^B^	0.81 ^A^	0.003	0.003	0.9643	0.0001	0.0582
Δ9−desat 20 ^15^	6.67	7.03	7.21	6.47 ^B^	7.47 ^A^	0.253	0.211	0.0968	0.0036	0.3511
Δ5+Δ6-desat ^16^	0.20	0.19	0.20	0.20 ^A^	0.19 ^B^	0.003	0.002	0.7949	0.0005	0.9616
Elongase ^17^	0.26	0.26	0.26	0.27 ^A^	0.25 ^B^	0.003	0.002	0.8933	0.0001	0.4822
Elongase (18/16) ^18^	0.38	0.38	0.38	0.40 ^A^	0.36 ^B^	0.007	0.005	0.8841	0.0001	0.4679
Thioesterase ^19^	79.32	77.07	77.72	78.04	78.04	1.493	1.272	0.8192	0.7171	0.0097

^1^ SEM: standard error of the mean of dietary treatment effect *n* = 18; ^2^ SEM: standard error of the mean of strain effect *n* = 36; ^3^ Differences were statistically significant when *p* < 0.05. ^a,b,ba,A,B^ Values with different superscript were statistically significant; ^4^ **∑**SAT: Sum of saturated fatty acids; ^5^**∑**MUFA: Sum of monounsaturated fatty acids; ^6^ **∑**PUFA: Sum of polyunsaturated fatty acids; ^7^ ∑n-6: Sum of n-6 polyunsaturated fatty acids; ^8^ ∑n-3: Sum of n-3 polyunsaturated fatty acids; ^9^ Hypocholesterolemic index = (C18:1 + C18:2 + C18:3 + C20:3+ C20:4 + C20:5 + C22:4 + C22:5 + C22:6)/(C14:0 + C16:0); ^10^ Thrombogenic index = (C14:0 + C16:0 + C18:0)/(0.5 × **∑**MUFA + 0.5 × **∑**n-6 + 3 × **∑**n-3 + **∑**n-3/**∑**n-6); ^11^Δ9 − desaturase index = (C14:1 + C16:1 + C18:1)/C14:0 + C14:1 + C16:0 + C16:1 + C18:0 + C18:1); ^12^ Δ9 − desaturase index 14 = (C14:1)/(C14:1 + C14:0); ^13^ Δ9 − desaturase index 16 = (C16:1n-7)/(C16:1n-7 + C16:0); ^14^ Δ9 − desaturase index 18 = (C18:1)/(C18:1 + C18:0); ^15^ Δ9 − desaturase index 20 = (C20:1)/(C20:1 + C20:0); ^16^ Δ5+ Δ6-desaturase = (C20:4n-6 + C20:5n-3 + C22:5n-3 + C22:6n-3)/(C18:2n-6 + C18:3n-3 + C20:4n-6 + C20:5n-3 + C22:5n-3 + C22:6n-3); ^17^ Elongase (18,20,22) index = C18:0 + C22:4n-6 + C22:5n-3/(C18:0 + C22:4n-6 + C22:5n-3 + C16:0 + C20:4n6 + C20:5n-3); ^18^ Elongase (18/16) index = C18:0/C16:0; ^19^ Thioesterase index = C16:0/C14:0.

**Table 7:** Effect of dietary spirulina and strain of laying hens (White Leghorn: WL or Rhode Island Red: RIR) on the iron-induced lipid oxidation (mmol MDA/kg) of yolk at 0, 60, 90 and 180 min of incubation.

	Dietary Treatment			Strain Effect		SEM ^1^	SEM ^2^	^3^*p* Treatment	*p* Strain	*p* Treatment × Strain
	CONTROL	1%	3%	WL	RIR					
**0 min**	0.036^b^	0.075^a^	0.07^a^	0.068	0.054	0.010	0.008	0.0209	0.2704	0.4981
**60 min**	0.237	0.266	0.273	0.245	0.272	0.017	0.015	0.1658	0.2809	0.3688
**90 min**	0.289	0.291	0.335	0.275	0.332	0.025	0.021	0.2338	0.0947	0.8427
**180 min**	0.29	0.313	0.386	0.327	0.333	0.019	0.016	0.1173	0.9467	0.2935

^1^ SEM: standard error of the mean of dietary treatment effect; ^2^ SEM: standard error of the mean of strain effect; ^3^ Differences were statistically significant when *p* < 0.05. Values with different superscript were statistically significant.

**Table 8:** Regression equations between different parameters of quality traits and yolk composition.

Variable y	Intercept	S.D. ^1^	Slope	S.D.	Variable x	r	R ^2^	*p* Linear
% Fat	63.123 ± 67.819 ± −5.399 ± 1.567 ± 0.976 ±	5.19	−0.649 ± −4.826 ± 7.443 ± −0.002 ± 2.973 ±	0.10	Humidity	−0.72	51.4	0.0001
CIE L*		2.50		1.39	Retinol	−0.46	21.0	0.0012
CIE a*		3.91		2.17	Retinol	0.49	23.7	0.0015
Total TBARS		0.14		0.00	α-tocopherol	−0.42	17.3	0.0129
Total TBARS		0.11		1.26	C22:5n-3	0.39	14.8	0.0245
Total TBARS	0.703 ±	0.23	2.508 ±	1.07	C22:4n-6	0.38	14.6	0.0256
SAT ^2^	41.469 ±	1.62	−2.542 ±	0.90	Retinol	−0.38	14.7	0.0072
C18:0	13.422 ±	0.84	−1.931 ±	0.47	Retinol	−0.52	26.7	0.0002
Δ9−des 18 ^3^	0.7348 ±	0.02	0.034 ±	0.01	Retinol	0.47	21.8	0.0008
Elongase ^4^	0.3148 ±	0.01	−0.031 ±	0.01	Retinol	−0.51	26.0	0.0002

^1^ S.D.: standard deviation; ^2^ SAT: Sum of satturated fatty acids; ^3^ Δ9 − desaturase index 18 = (C18:1)/(C18:1 + C18:0); ^4^ Elongase (18, 20, 22) index = C18:0 + C22:4n-6 + C22:5n-3/ (C18:0 + C22:4n-6 + C22:5n-3 + C16:0 + C20:4n6 + C20:5n-3). L* (luminosity); a* (redness).

## Data Availability

Not applicable.
